# Hypoxic-Ischemic Encephalopathy-Induced Seizure in an 11-Year-Old Female

**DOI:** 10.7759/cureus.16606

**Published:** 2021-07-24

**Authors:** Krunal Pandav, Angela Ishak, Farah Chohan, Omoyeme Edaki, Jonathan Quinonez, Samir Ruxmohan

**Affiliations:** 1 Division of Research & Academic Affairs, Larkin Community Hospital, South Miami, USA; 2 Division of Research & Academic Affairs, Larkin Health System, South Miami, USA; 3 Osteopathic Neuromusculoskeletal Medicine, Larkin Community Hospital, South Miami, USA; 4 Neurology, Larkin Community Hospital, South Miami, USA

**Keywords:** ischemia, brain hypoxia, hypoxia, encephalopathy, seizure, brain

## Abstract

Hypoxic-ischemic encephalopathy (HIE) typically manifests in the neonatal period. The degree of hypoxia following intrapartum asphyxia determines the structural changes in the brain, which can cause functional deficits in the affected child leading to developmental deficits and recurrent seizures. Management requires physical therapy, occupational therapy, and anti-seizure medications. We present a rare case of an 11-year-old female with a past medical history of epilepsy and cerebral atrophy secondary to hypoxic injury at birth. The patient presented to the hospital following a witnessed seizure and loss of consciousness for one hour. Given the past medical history and clinical findings, it was determined that a mild-to-moderate encephalopathic process resulted in a lower seizure threshold. HIE can manifest beyond the neonate years mainly due to the structural changes within the brain. Therefore, it is essential to understand aspects of HIE beyond the neonate years to manage this condition for a better patient outcome.

## Introduction

Hypoxic-ischemic encephalopathy (HIE) is a significant and frequent cause of acute mortality and chronic neurological disability in neonates [1]. HIE usually manifests in the neonatal period and often leads to cerebral palsy, mental retardation, learning disability, and epilepsy in the survivors [2,3]. Around 15% to 20% of the asphyxiated infants exhibiting HIE do not survive the newborn period. From the surviving population, around 25% show signs of permanent neuro-psychological damage [4]. HIE can also manifest in late childhood and adulthood. It arises in such cases due to global hypoperfusion due to cardiac arrest, drowning, or hypotension [5]. A retrospective study by Muttikkal et al. showed that the prognosis of adults with HIE is relatively poor with only three patients in their study exhibiting signs of clinical improvement [6].

Neurological injury resulting from hypoxia-ischemia may occur due to either or both processes; neuronal necrosis and apoptosis. The main pathological mechanism underlying intrapartum hypoxia-ischemia is impaired cerebral blood flow resulting from an interruption in placental blood flow and gas exchange. This phenomenon is often termed asphyxia or severe fetal academia [7]. Treatment strategies for HIE have been mostly limited to supportive intensive care. However, during recent times, whole-body hypothermia has emerged as the standard of care [8]. Whole-body hypothermia has favorable effects on multiple factors contributing to brain injury, including excitatory amino acids, the cerebral energy state, cerebral blood flow and metabolism, nitric oxide production, and apoptosis. It has also been shown to reduce the risk of mortality and disability in infants with moderate or severe HIE [9]. 

With this, we present a case of an 11-year-old developmentally delayed female with a past medical history of epilepsy and cerebral atrophy secondary to hypoxic insult to the brain at the time of birth. Informed consent was obtained from the patient and we followed the CARE guidelines.

## Case presentation

**Figure 1:**
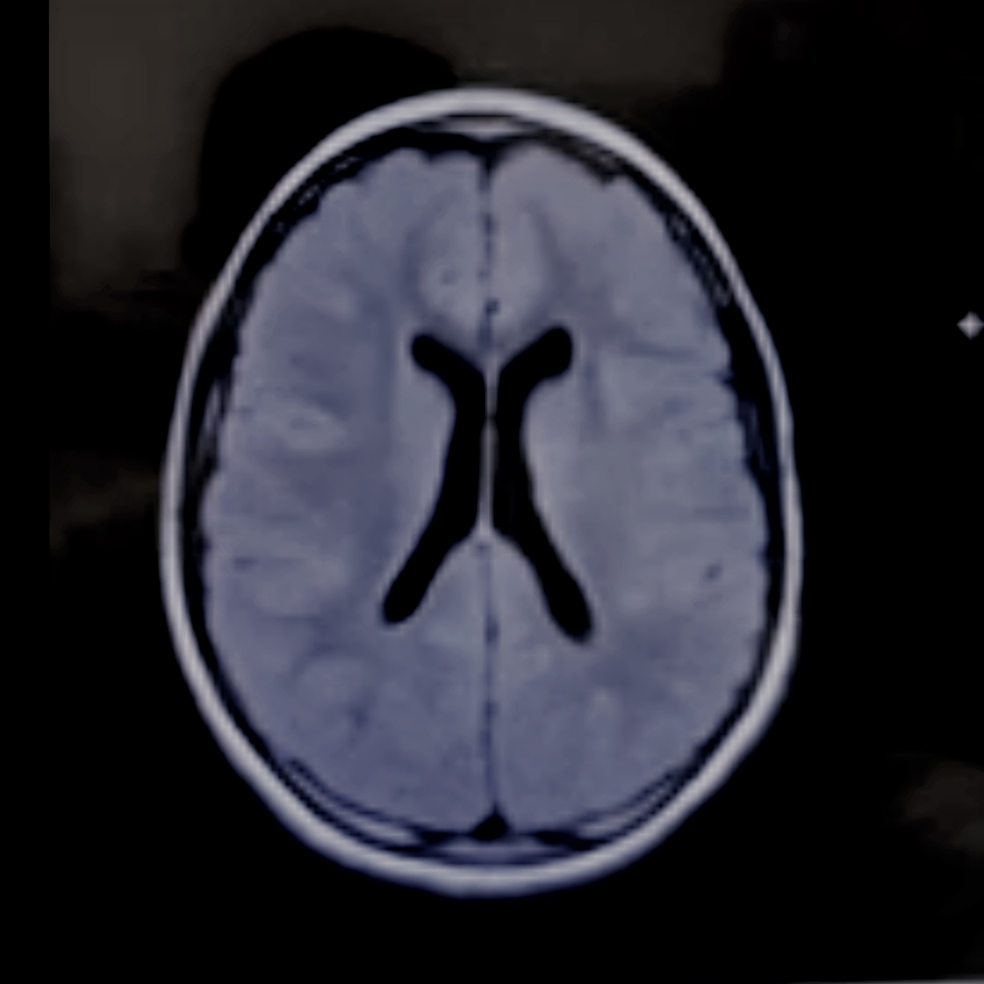
MRI Brain Axial View No evidence of mass effect, midline shift or intracranial hemorrhage. Hypoplastic appearance of the corpus and splenium. No ventriculomegaly.

**Figure 2:**
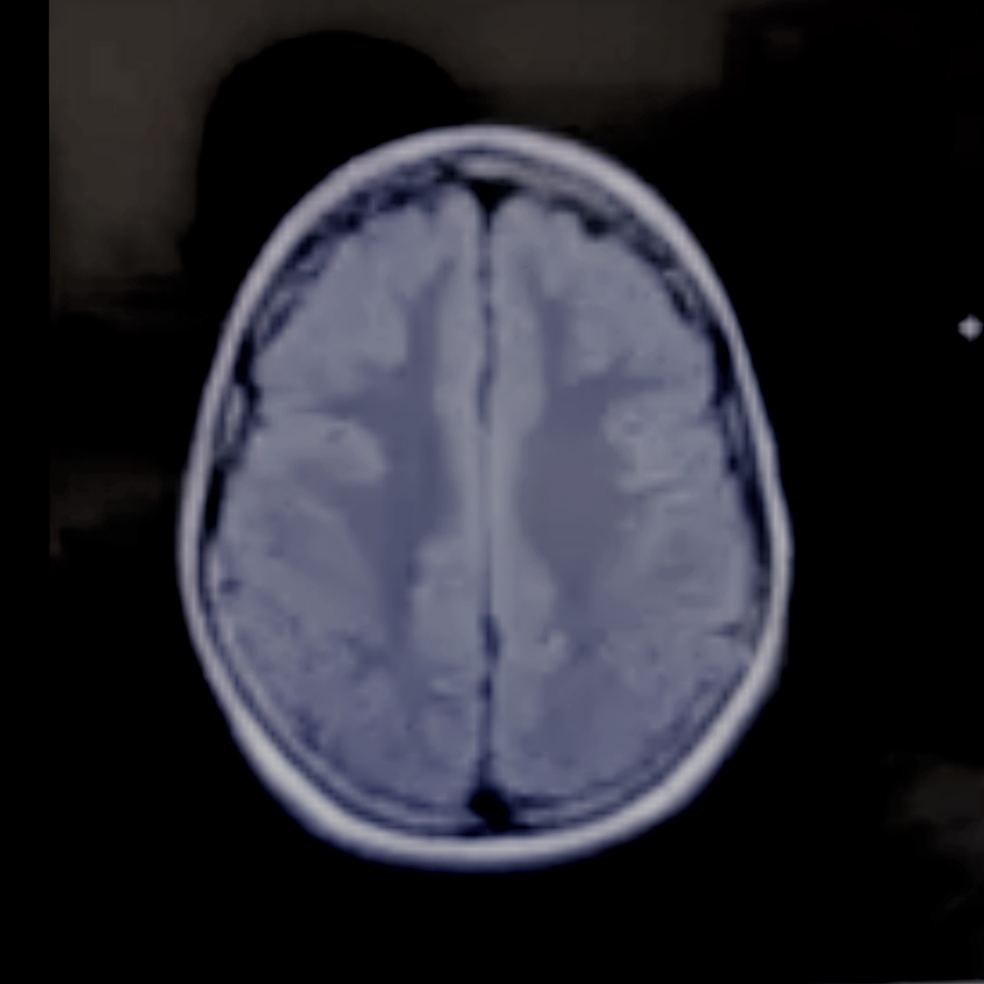
MRI Brain Axial View No evidence of mass effect, midline shift or intracranial hemorrhage. Hypoplastic appearance of the corpus and splenium. No ventriculomegaly.

The patient was born at 36-weeks’ gestation age and was delivered via Cesarean section to a mother with preeclampsia. It was later discovered that the patient had cerebral atrophy secondary to hypoxic injury at birth. The patient exhibited global developmental delays in which they started to walk at 26 months and exhibited "articulation issues'' during the conversation. In addition, the patient also experienced febrile seizures between the ages of one and five. The seizures progressed to generalized tonic-clonic seizures in several years. The patient’s mother and grandmother described it as “staring/unresponsive episodes.” The patient was being administered oral levetiracetam (Keppra) 4 ml twice daily, oral lorazepam (Ativan), and rectal diazepam (Diastat) for seizures. Despite the ongoing management, the patient experienced seizures leading to recurrent hospitalizations. Prior imaging studies during their workup included video encephalograms (vEEG), a CT scan of the head without contrast, and an MRI of the brain with and without contrast (Figures 1-2).

The imaging studies are summarized in Table 1.

**Table 1:** Summary of the diagnostic studies done vEEG = Video electroencephalogram; MRI: Magnetic resonance imaging

Type of diagnostic study	Year of the study	Significant findings
vEEG	2017	Abnormal awake/sleep vEEG – diffuse cortical dysfunction found.
vEEG	2018	Abnormal awake vEEG study – moderately slow background with intermittent bifrontal delta slowing indicating a mild-moderate encephalopathic process.
MRI of the brain with and without contrast	2018	Hypophasic appearance of the splenium and posterior corpus callosum body (Figures 1-2).
vEEG	2019	Abnormal awake vEEG study – rare intermittent diffuse background slowing over the bifrontal regions and frequent epileptiform discharges over the left temporal and right posterior head regions.

The patient presented to the hospital because she began to “stare ahead” while in the presence of her grandmother. The grandmother noticed the patient exhibiting “rolled eyes,” jaw clenching, as well as unresponsiveness. No other abnormal movements were noticed. The patient had several non-bloody emesis episodes. The caregiver administered oral Ativan, but the patient showed signs of dysphagia.

Upon presentation to the hospital, the patient had bowel incontinence and an additional emesis episode with a low-grade temperature of 100.1F; 2 mg IV Ativan followed by 20 mg/kg IV Keppra bolus were administered. The following day, a physical examination was unremarkable; however, the neurology examination revealed poor attentiveness and decreased language/comprehension. Laboratory testing was unremarkable. A CT scan of the head was performed, which showed no intracranial abnormalities. On improvement, the patient was discharged with a seizure rescue plan of Ativan 2 mg if a seizure lasted more than three minutes; Keppra was also increased to 500 mg twice daily due to a lower seizure threshold in the setting of recurrent emesis episodes and a low-grade temperature. The patient was instructed to follow up with the neurologist in 2-4 weeks.

## Discussion

HIE is a type of neonatal encephalopathy (NE) that is mainly due to oxygen deprivation to the neonate's brain. The presentation of HIE can range from depression of tone and reflexes, difficulty initiating and maintaining respiration to subnormal levels of consciousness and seizures [10]. The underlying pathophysiology of these presentations involves oxidative stress, glutaminergic excitotoxicity, failure of mitochondrial energy production, as well as apoptosis [11]. Based on the presentation of our patient, she may have been classified as having moderate-to-severe HIE at infancy which predisposed her to develop cerebral atrophy and other complications during her childhood. About 16% of neonates with moderate-severe HIE will develop epilepsy. It is well established that male infants with HIE are at an increased risk of suffering long-term deficits as compared to females [12], making the presentation in this case unusual. The exact mechanisms of the development of epilepsy in these children are not well understood. A study suggested that early MRI injuries are associated with the development of epilepsy in such patients [13]. Structural brain changes could lead to lower seizure thresholds in affected children, increasing their likelihood of developing recurrent seizures. This could especially occur in the presence of other provoking factors that can include emesis and low-grade fevers, such as in this patient's case.

The patient's seizures progressed over time to become generalized tonic-clonic seizures. This can be corroborated by the MRI findings, which showed hypoplastic changes in the splenium and posterior corpus callosum body. In addition, a recent literature review suggested that even though the corpus callosum does not act as a seizure center, it serves as a major pathway for the generalization of seizures [14]. This may provide some evidence for the presentation of seizures in our patient. The type of seizure exhibited by patients with HIE may vary, and the presentation and duration of these seizures will determine the type of anti-epileptic drug to utilize. In our case, the patient’s current combination of Keppra, Ativan, and Diastat proved insufficient in managing her epilepsy. The patient required a moderate increase in Keppra to 500 mg twice daily to control her seizures due to her lowered seizure threshold. Keppra was used as it has been documented as an effective drug as adjunctive therapy in partial seizures and has shown good efficacy in refractory mixed seizure disorders [15], such as in our case. Ativan’s main advantage is that it has shown significantly lessened seizure recurrence with mild side effects and is highly effective in treating prolonged seizures [16]. Due to the complex etiology of the patient's seizures, and low seizure threshold, a combination of drugs was deemed necessary to control the seizures with consistent follow-ups to tackle any other complications that could arise due to HIE.

## Conclusions

This case report describes a female adolescent patient who experienced epilepsy due to a "low seizure threshold" resulting from HIE aggravated by other provoking factors. HIE is an important condition for clinicians to consider as brain structural defects can manifest beyond the neonate years making seizure management in these patients complicated. Extensive follow-up is important as patients with HIE will develop a "low seizure threshold" and require further management than the general population.
